# Hyphal Growth and Conidia Germination Are Induced by Phytohormones in the Root Colonizing and Plant Growth Promoting Fungus *Metarhizium guizhouense*

**DOI:** 10.3390/jof9090945

**Published:** 2023-09-19

**Authors:** Iván Horacio Piña-Torres, Fabiola Dávila-Berumen, Gloria Angélica González-Hernández, Juan Carlos Torres-Guzmán, Israel Enrique Padilla-Guerrero

**Affiliations:** Departamento de Biología, División de Ciencias Naturales y Exactas, Universidad de Guanajuato, Noria Alta s/n, Guanajuato 36050, Mexico; ih.pina@ugto.mx (I.H.P.-T.); fabidabe@live.com.mx (F.D.-B.); gonzang@ugto.mx (G.A.G.-H.); torguz@ugto.mx (J.C.T.-G.)

**Keywords:** phytohormone, fungus–plant interaction, germination conidia promotion, hyphal growth promotion, root colonization, plant growth promotion, sorghum

## Abstract

Beneficial associations are very important for plants and soil-dwelling microorganisms in different ecological niches, where communication by chemical signals is relevant. Among the chemical signals, the release of phytohormones by plants is important to establish beneficial associations with fungi, and a recently described association is that of the entomopathogenic ascomycete fungus *Metarhizium* with plants. Here, we evaluated the effect of four different phytohormones, synthetic strigolactone (GR24), sorgolactone (SorL), 3-indolacetic acid (IAA) and gibberellic acid (GA_3_), on the fungus *Metarhizium guizhouense* strain HA11-2, where the germination rate and hyphal elongation were determined at three different times. All phytohormones had a positive effect on germination, with GA_3_ showing the greatest effect, and for hyphal length, on average, the group treated with synthetic strigolactone GR24 showed greater average hyphal length at 10 h of induction. This work expands the knowledge of the effect of phytohormones on the fungus *M. guizhouense*, as possible chemical signals for the rapid establishment of the fungus–plant association.

## 1. Introduction

Associations are important for all organisms on earth to maintain life. Plants, for example, form associations in the rhizosphere with microorganisms and organisms such as animals and other plants for their adequate assimilation of nutrients [1]. Such associations are carried out by the exchange of chemical signals between the organisms, as is the case for plants, which release, through their roots, a wide range of compounds that participate in the attraction of beneficial organisms and the formation of mutualistic associations in the rhizosphere, in which complex biological and ecological processes occur [1,2]. Among the most important compounds used by plants are sugars, amino acids, proteins, fatty acids, aromatic, aliphatic, phenolic compounds, plant growth regulating enzymes and other secondary metabolites [1].

One of the most important beneficial associations occurs between plant roots and fungi, forming a mycorrhizal symbiosis [3]. Mycorrhizal associations involve improved uptake of soil nutrients such as nitrogen and phosphorus through fungal hyphae into plant roots or seeds, improved resistance to stress [3,4], resistance to pathogens [5], tolerance to heavy metals [6] and improved moisture retention [7], which also influences soil structure and supports the multifunctionality of the ecosystem [8]. The fungus benefits from organic carbon compounds coming from the plant, and thus, the fungus can take advantage of these compounds to develop [3].

As mentioned above, there are chemical substances that help in the signaling and establishment of mycorrhizal symbiosis, some of which are phytohormones, such as auxins, cytokinins, ethylene and strigolactones, which participate in developmental and other metabolic processes of plants [9]. For example, cytokinins can be produced by mycorrhizal fungi, favoring the association and activating plant defense responses for future attacks [10,11]. Reportedly, the fungi *Laccaria bicolor*, *Tuber borchii*, *T. melanosporum* and recently a fungus classified as endophytic entomopathogenic, *Metarhizium robertsii*, produce 3-indolacetic acid (IAA), which is a type of auxin that modifies root structure, increasing root growth and the number of secondary roots. Furthermore, *L. bicolor* produces ethylene, which activates the IAA synthesis pathway, increasing root growth and improving the association with the fungus [11,12].

In addition, phytohormones can modify the metabolism of the fungus. In the early stages of host recognition, the hyphae of the mycorrhizal fungus show extensive branching in the vicinity of the host roots and the establishment of symbiosis [13]; such is the case for cytokinins, which stimulate the growth and branching of hyphae [10]. Auxins influence the colonization of arbuscular mycorrhizal fungi and can promote arbuscule development, while gibberellin together with DELLA proteins regulate arbuscule formation in plant cells [14].

Another recently discovered family of phytohormones is the strigolactones (SLs), which are plant carotenoid derivatives involved in seed germination, suppression of shoot branching and formation of root architecture. Under nitrogen and phosphorus-limiting conditions, these phytohormones are released in large quantities by the plant into the rhizosphere to establish mycorrhizal associations. This family of SLs stimulates the development and further branching of hyphae by activating the mitochondria of arbuscular mycorrhizal fungi and favoring the establishment of the mycorrhizal association. The spores of *Rhizophagus irregularis* treated with a synthetic analog of SL, called GR24, stimulated the production of some chitin oligomers, tetramers and pentamers, by 38 and 69 times [13,14,15]. This family of SLs phytohormones are also detected by parasitic plants such as the genus *Striga* [16] and phytopathogenic fungi such as *Fusarium oxysporum* [17] and can strongly affect agricultural crops. One of the plants that has been found to produce SLs is sorghum (*Sorghum bicolor*), which produces sorgomol, strigol, orobanchol and sorgolactone to establish mycorrhizal associations [18,19,20,21].

The fungal genus *Metarhizium*, is better known for its parasitic symbiosis with pest insects [22], plays an important ecological role related to the rhizosphere and plant roots. The ability to compete in the rhizosphere against other microorganisms and to become attached to plant roots has been shown [23,24], establishing complete root colonization of bean and switchgrass [25,26]. In 2012, Behie and collaborators tested the ability of this fungus to transfer nitrogen from infected *Galleria mellonella* larvae injected with ^15^N to the plant. After 14 days, the insect-derived nitrogen constituted 28% to 32% of the nitrogen content in beans and switchgrass, respectively, representing a large percentage of ^13^N incorporation by the plants [27].

Subsequently, the reciprocity and association between *Metarhizium* and the plant was determined using ^13^CO_2_, which was used by the plant to synthesize its own sugars. *Metarhizium*-colonized roots receive the carbon photosynthetically fixed by the plant, and *Metarhizium* is able to metabolize this carbon for use in metabolic processes [28]. In 2021, S. Hu and M. J. Bidochka [26] demonstrated that exogenous application of abscisic acid on the root of *Phaseolus vulgaris* decreases the colonization of *Metarhizium*, demonstrating the importance of phytohormones in the interaction with plants. However, unlike other plant–fungal associations, such as arbuscular mycorrhizae and ectomycorrhizae [3], in the genus *Metarhizium*, the chemical communication and the compounds involved from the plant to the fungus have not yet been determined to establish a beneficial association.

The objective of this work is to identify the effects of *Metarhizium guizhouense* HA11-2 interaction with sorghum plants as a growth promoter and colonization, as well as to clarify the phenotypic changes in conidial germination and hyphal growth, in the presence of different types of phytohormones.

## 2. Materials and Methods

### 2.1. Growth Conditions

*M. guizhouense* strain HA11-2 was isolated from soil in Guanajuato, Mexico, and belongs to the collection of the Laboratory of Molecular Genetics of Fungi, Department of Biology, Division of Natural and Exact Sciences, University of Guanajuato. Phylogenetic analysis was performed using a 5’ region of the translation elongation factor 1-alpha (EF-1α) gene and internal transcribed spacer (ITS) sequence according to Bishop et al., 2009 [29]. EF-1α and ITS sequences were deposited in GenBank under accession numbers OQ800927 and OQ784854, respectively. The *M. guizhouense* transformant strain HA11-2-GFP was generated according to transformation protocols with *Agrobacterium* [30,31]. Strains were routinely cultured on M100-2N solid medium [Culture medium M-100 [30], supplemented with 22.4 mM ammonium nitrate (NH_4_NO_3_) (Karal^®^, León, México)] and incubated at 28 °C with a photoperiod of 16 h light/8 h dark for ten days until conidiation.

### 2.2. Collecting Conidia

To collect *M. guizhouense* conidia, 0.1% Triton X-100 solution (Sigma^®^, Saint Louis, MO, USA) was used on the solid medium where *M. guizhouense* grew, and conidia were scraped, collected and filtered with a synthetic fiber mesh to remove the mycelium. Three washes were performed with 30 mL of 0.01% Triton X-100. Finally, the conidia were suspended in a volume of 20 mL, and the concentration was determined by counting with a hemocytometer (Hausser Scientific^®^, Horsham, PA, USA). The conidial solution was stored at 4 °C for later use the next day in each of the experiments. The process was repeated in each of the experiments to use fresh conidia.

### 2.3. Evaluation of Sorghum Growth Promotion by Metarhizium

Sorghum (*S. bicolor*) seeds were surface sterilized with 80% alcohol solution for 1 min, 4% sodium hypochlorite (NaClO) (Cloralex^®^, Monterrey, México) for 2 min, three washes with sterile distilled water for 1 min each, and subsequently dried and stored until use. Using a solution of 1 × 10^8^ conidia/mL of *M. guizhouense* strain HA11-2, the conidial pellet was recovered and resuspended in 250 µL of a 0.5% carboxymethylcellulose sodium (Sigma^®^, Saint Louis, MO, USA) solution. The resulting conidial suspension was added to a tube of 50 mL containing 20 sorghum seeds and mixed in vortex for 1 min, allowing the seeds to dry for 5 min. Six replicates were performed with sixteen sorghum seeds treated with *Metarhizium* conidia sown in peat moss (Kekkilä Professional^®^, Vantaa, Finland) as substrate in plastic pots of 15 × 8 cm and initially irrigated with 100 mL of water. As an experimental control, sorghum seeds were treated in the same way, excluding the use of *Metarhizium* conidia. The pots were incubated in a greenhouse for 30 days and watered with 100 mL of distilled water every 48 h. After this time, plant length and dry weight were measured.

### 2.4. Sorghum Root Colonization by M. guizhouense

The sterilized sorghum seeds were placed in water-agar medium 15 g of (Bioxon^®^, Cuautitlán Izcalli, México) bacteriological agar per liter of distilled water. They were incubated at 28 °C in a photoperiod of 16 h of light/8 h of darkness, and when the roots grew three centimeters, they were immersed in a *M. guizhouense* HA11-2-GFP solution of 1 × 10^8^ conidia/mL in 0.01% Triton-X for 5 min for adherence of the conidia. Plants were placed on fresh water-agar medium plates and incubated at 28 °C with a 16 h light/8 h dark photoperiod for subsequent observation by fluorescence microscopy.

### 2.5. Phytohormone Assay

Twenty microliters of a solution containing 1 × 10^6^ conidia/mL of *M. guizhouense* strain HA11-2 was placed 1.5 cm from the center on a plate with fresh solid water agar medium. Then, a 20 mm diameter filter paper disk (previously sterilized) was placed 1.5 cm from the center, toward the opposite side of the *Metarhizium* drop, where a 20 µL drop of a solution of any of the following phytohormones was placed: Synthetic strigolactone rac-GR24 (GR24) (ChemPep^®^, Wellington, FL, USA), (+−)-sorgolactone (SorL) (ChemPep^®^, Wellington, DC, USA), 3-indolacetic acid (IAA) (Sigma^®^, Saint Louis, MO, USA) and gibberellic acid (GA_3_) (Sigma^®^, Saint Louis, MO, USA), at different concentrations ranging from 1 × 10^3^ M to 1 × 10^6^ M. They were incubated at 28 °C in total darkness for 6, 8 or 10 h. Nine replicates were performed for each treatment. For phytohormones GR24 and SorL, DMSO (Sigma^®^, Saint Louis, MO, USA) was used as the solvent, and for GA_3_ and IAA, absolute EtOH (molecular biology grade, Karal^®^, León, México) was used.

Once the incubation time had elapsed, a 1 cm^2^ square of agar was cut where the drop of *M. guizhouense* solution had been placed; this square was placed on an object holder with a cover slip on top to later visualize and take photos under a bright-field microscope. From the photos, germination rate and length of the hyphae were analyzed.

### 2.6. Microscopic Analysis

Photographs of conidia were taken on a Zeiss Primo Star (Carl Zeiss^®^, Jena, Germany.) brightfield microscope with a Primo Plan-ACHROMAT 40×/0.65 lens using the AxioCam ICc 1 camera and ZEN 3.4 Blue edition software from Carl Zeiss (Jena, Germany) for the corresponding hyphal measurements, adjusting the scale with a micrometer. Photographs of the root colonized with *M. guizhouense* strain HA11-2-GFP were taken using a Zeiss Axio Zoom V.16 fluorescence stereomicroscope with Plan Neo Fluar Z 1×/0.25 FWD 56 mm lens 40× zoom.

### 2.7. Statistical Analysis

GraphPad Prism v. 9.0 (GraphPad Software, Boston, MA, USA) was used for statistical analysis. The data were evaluated by identifying outliers using the ROUT method (Q = 1%), and then the Komogorov–Smirnov (KS) test and Shapiro-Wilk test were used to determine whether they were normally distributed. For the pairwise comparison analyses, the two-tailed *T* test was used for normally distributed data, and the Mann-Whitney U test was used for nonnormally distributed data. For multiple comparison analysis, one-way ANOVA was used for normally distributed data (parametric data) with Tukey’s multiple comparison test, and the Kruskal-Wallis test was used for nonnormally distributed data (nonparametric data) with Dunn’s multiple comparison test. All analyses were performed with a 95% confidence interval variance. The RStudio package (Version 2022.12.0+353) was used for the heatmap graphics.

## 3. Results

### 3.1. Metarhizium guizhouense HA11-2 Is a Growth Promoter and Root Colonizer of Sorghum

**Figure 1:**
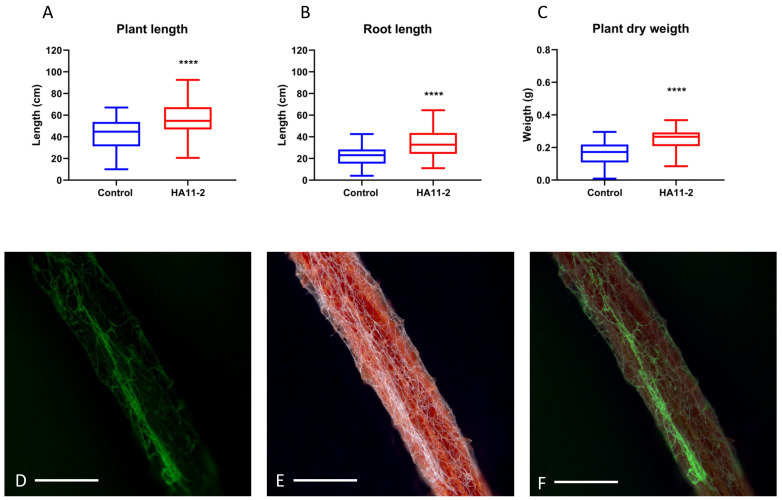
Sorghum growth promotion and root colonization by *M. guizhouense* strain HA11-2. (**A**) Length of the whole sorghum plant; Student’s *t*-test, *p* < 0.0001, *t* = 4.26, *df* = 84. (**B**) Sorghum root length; Student’s *t*-test, *p* < 0.0001, *t* = 4.67, *df* = 79. (**C**) Sorghum plant dry weight; Mann–Whitney U test, *p* < 0.0001, *U* = 351.5. GP: 0.1234 (ns), <0.0001 (****). (**D**) Green-field fluorescence image of root colonization by the fungal mycelium. (**E**) Bright-field image of root colonization (red) by the fungal mycelium (white). (**F**) Splicing of the florescence image and the bright-field image of root colonization by the fungal mycelium. White scale bar distance 0.5 mm.

The growth-promoting capacity of sorghum was evaluated under semicontrolled greenhouse conditions, and there was a significant difference between plants treated with *M. guizhouense* HA11-2 and those that were not treated. The average total plant length (Figure 1A) of the control plants was 42.25 cm (std. deviation: 15.43) and that of the plants treated with the fungus was 56.09 cm (std. deviation: 14.69); the average root length (Figure 1B) of the control plants was 22.22 cm (std. deviation: 9.73) and that of the treated plants was 33.19 cm (std. deviation: 11.9); and the average plant dry weight (Figure 1C) of the control plants was 0.16 g (std. deviation: 0.0717) and that of the treated plants was 0.24 g (std. deviation: 0.0705).

Furthermore, the capacity to colonize the sorghum root was also observed. Strain HA11-2 was found to have the ability to colonize sorghum plant roots in nutrient-deficient medium (water–agar), using a strain expressing green fluorescent protein (HA11-2-GFP) (Figure 1). It is clearly observed that the mycelium of the fungus was white around the root, forming network-like structures. This could be seen in the bright field image (Figure 1E) and in the fluorescence field (Figure 1D). Figure 1F shows the brightfield and fluorescence field merged.

### 3.2. Phytohormones Induce Germination of Metarhizium guizhouense HA11-2

**Figure 2:**
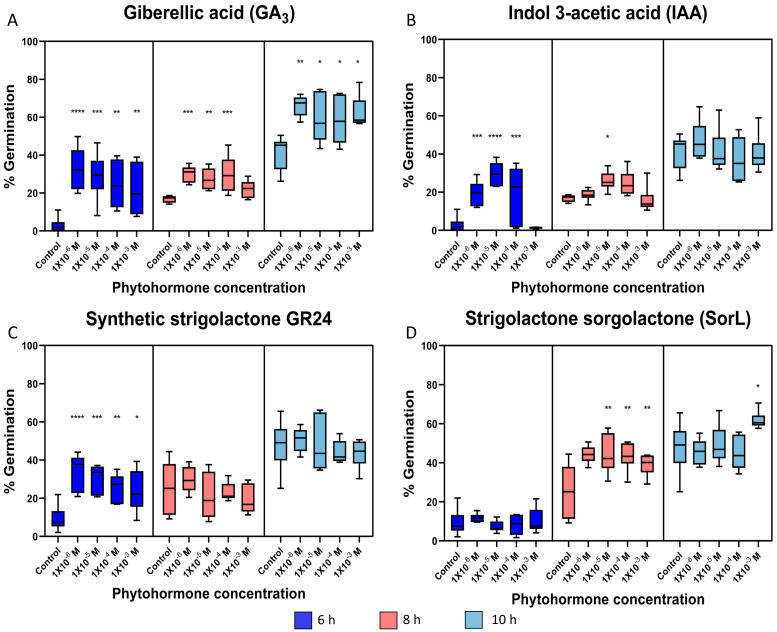
Germination rate graphs. The graphs are divided into the three times marked in blue, 6 h; pink, 8 h; and sky blue, 10 h. Asterisks mark those with significant differences vs. control. (**A**) Gibberellic acid (GA_3_): 6 h; ANOVA one-way test, *p* ≤ 0.0001, *F*(4.31) = 11.67. 8 h; ANOVA one-way test, *p* ≤ 0.0001, *F*(4.31) = 10.32. 10 h; ANOVA one-way test, *p* = 0.0029, *F*(4.25) = 5.37. (**B**) 3-indole acetic acid (IAA): 6 h; ANOVA one-way test, *p* ≤ 0.0001, *F*(4.31) = 20.44. 8 h; Kruskal-Wallis test, *p* = 0.0008, *H*(4.36) = 18.91. 10 h; ANOVA one-way test, *p* = 0.5601, *F*(4.25) = 0.76. (**C**) Synthetic strigolactone rac-GR24 (GR24): 6 h; ANOVA one-way test, *p* ≤ 0.0001, *F*(4.31) = 12.03. 8 h; Kruskal-Wallis test, *p* = 0.4215, *H*(4.36) = 3.89. 10 h; ANOVA one-way test, *p* = 0.7278, *F*(4.31) = 0.5113. (**D**) Strigolactone (+−)-sorgolactone (SorL): 6 h; ANOVA one-way test, *p* = 0.6070, *F*(4.31) = 0.69. 8 h; ANOVA one-way test, *p* = 0.0004, *F*(4.31) = 6.94. 10 h; ANOVA one-way test, *p* = 0.0276, *F*(4.31) = 3.15. GP: 0.1234 (ns), 0.0332 (*), 0.0021 (**), 0.0002 (***), <0.0001 (****). Only those that were positively significant vs. control are shown.

A positive change in conidia germination was observed with all of the evaluated phytohormes (Supplementary Figure S1A,B). With the phytohormone GA_3_, a significant difference was observed at all times evaluated (Figure 2), except for the 8 h time at a concentration of 1 × 10^−3^ M, although a positive trend is observed (Figure 2A). On average, there was a higher germination rate with the lowest concentration of 1 × 10^−6^ M at the three times (Supplementary Figure S2 and Table S1). For the phytohormone IAA (Figure 2B), the percentage of germination clearly increased at early times, from the concentration of 1 × 10^−3^ to 1 × 10^−6^ M, with the highest percentage at the concentration of 1 × 10^−5^ M. At 8 h, the tendency of increased germination was observed, but statistically, there was a significant difference only with the 1 × 10^−5^ M concentration. The highest germination rate was observed at 6 h with the 1 × 10^−5^ M concentration (Supplementary Figure S2 and Table S1).

The other two phytohormones evaluated, SorL and GR24 (Figure 2C,D and Supplementary Figure S2), correspond to the strigolactone group. For GR24, at 6 h, a significant increase in germination was observed at all the concentrations used, with the lowest concentration of 1 × 10^−6^ M with a higher rate (Figure 2C, Supplementary Figure S2 and Table S1). For SorL, at 8 h, a significant increase in germination was observed, except at the 1 × 10${}^{-6}$ M concentration, with a higher rate observed at the 1 × 10${}^{-5}$ M concentration. In addition, with SorL, at 10 h, a significant difference was observed only with 1 × 10${}^{-3}$ M (Figure 2D, Supplementary Figure S2 and Table S1).

### 3.3. Phytohormones Induce the Growth of M. guizhouense HA11-2 Hyphae

In addition to determining the percentage of germination with the different phytohormones, their participation in the growth of the hyphae of the conidia of *M. guizhouense* strain HA11-2 was also evaluated. Similarly, the four phytohormones were evaluated three times, as mentioned above.

**Figure 3:**
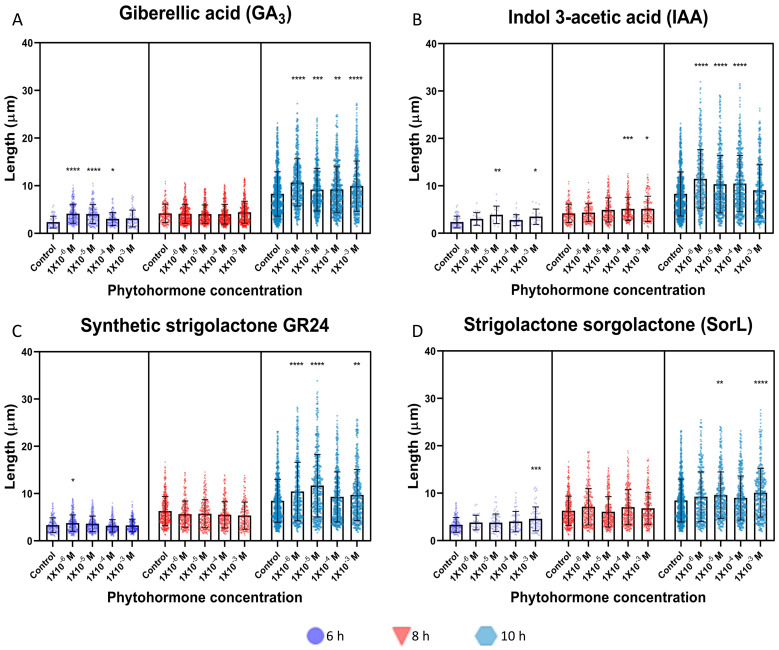
Hyphal length graphs. The graphs are divided into the three times, represented by a purple circle, 6 h; red triangle, 8 h; and sky blue hexagon, 10 h. Asterisks mark those with significant differences vs. control. (**A**) Gibberellic acid (GA_3_): 6 h; Kruskal-Wallis test, *p* ≤ 0.0001, *H*(4.718) = 83.21. 8 h; Kruskal-Wallis test, *p* = 0.0610, *H*(4.1720) = 9.00. 10 h; Kruskal-Wallis test, *p* ≤ 0.0001, *H*(4.3017) = 95.61. (**B**) 3-indole acetic acid (IAA): 6 h; Kruskal-Wallis test, *p* = 0.0007, *H*(4.152) = 19.37. 8 h; Kruskal-Wallis test, *p* = 0.0003, *H*(4.912) = 21.36. 10 h; Kruskal-Wallis test, *p* ≤ 0.0001, *H*(4.2713) = 102.4. (**C**) Synthetic strigolactone rac-GR24 (GR24): 6 h; Kruskal-Wallis test, *p* ≤ 0.0001, *H*(4.1313) = 24.21. 8 h; Kruskal-Wallis test, *p* = 0.0005, *H*(4.1285) = 20.10. 10 h; Kruskal-Wallis test, *p* ≤ 0.0001, *H*(4.2388) = 70.93. (**D**) Strigolactone (+−)-sorgolactone (SorL): 6 h; Kruskal-Wallis test, *p* = 0.0015 *H*(4.416) = 17.57. 8 h; Kruskal-Wallis test, *p* = 0.0020, *H*(4.1528) = 16.95. 10 h; Kruskal-Wallis test, *p* ≤ 0.0001, *H*(4.2121) = 31.64. GP: 0.1234 (ns), 0.0332 (*), 0.0021 (**), 0.0002 (***), <0.0001 (****). Only those that were positively significant vs. control are shown.

As in germination, hyphal growth was positively affected by all the phytohormones evaluated (Supplementary Figure S1C–F). For the phytohormone GA_3_, a significant effect was observed at 6 h and 10 h of induction, from the concentration of 1 × 10${}^{-4}$ M to 1 × 10${}^{-6}$ M for 6 h and at all concentrations at 10 h. The lowest concentration of 1 × 10${}^{-6}$ M showed the greatest effect, having a mean length of 10.70 µm, which was 2.41 µm greater than that of the control (Figure 3A, Supplementary Figure S3 and Table S2). For the phytohormone IAA, the effect was observed at all times evaluated, but not at all concentrations. At 6 h, the hyphal growth promoting effect was observed at concentrations of 1 × 10${}^{-3}$ M and 1 × 10${}^{-5}$ M, at 8 h of induction at 1 × 10${}^{-3}$ M and 1 × 10${}^{-4}$ M and at 10 h of induction from 1 × 10${}^{-4}$ M to 1 × 10${}^{-6}$ M. The highest growth was observed at 1 × 10${}^{-6}$ M on average of 11.47 µm, which was 3.18 µm more than that of the control and 0.77 µm more than that of GA_3_ (Figure 3B, Supplementary Figure S3 and Table S2).

Hyphal growth was induced by strigolactones GR24 and SorL at both 6 h and 10 h (Figure 3C,D, Supplementary Figure S3 and Table S2). For phytohormone GR24 at 6 h, only at the concentration of 1 × 10${}^{-6}$ M a significant difference was observed, and at 10 h, a significant difference was observed at the concentrations of 1 × 10${}^{-3}$ M, 1 × 10${}^{-5}$ M and 1 × 10${}^{-6}$ M. At 1 × 10${}^{-5}$ M, the highest mean length of 11.69 µm was the highest mean hyphal length of all phytohormones evaluated and was 3.29 µm more than that of the control (Figure 3C, Supplementary Figure S3 and Table S2). For the phytohormone SorL, at 6 h, a significant difference was observed only with a concentration of 1 × 10${}^{-3}$ M and at 10 h with concentrations of 1 × 10${}^{-3}$ M and 1 × 10${}^{-5}$ M with a mean hyphal length of 10.08 µm for the highest concentration (1 × 10${}^{-3}$ M), which was 1.621 µm more than that of the control (Figure 3D, Supplementary Figure S3 and Table S2).

## 4. Discussion

No organism is exempt from having associations with others, both beneficial and detrimental. One important beneficial association is that of plants with mycorrhizal fungi, which aid in nutrient exchange and root protection [3]. Such is the case for the entomopathogenic fungus *Metarhizium*, which is harmful to herbivorous insects and has been widely used in agricultural fields as a bioinsecticide, but has also been shown to be a root colonizer, plant growth promoter and protector of roots from phytopathogenic fungi [22,25,32,33,34,35]. Here, we demonstrate the root colonizing and plant growth promoting ability of *M. guizhouense* strain HA11-2 in sorghum (Figure 1). In mycorrhizal fungi, fast colonization is important because in this zone, nutrient exchange takes place. Some mycorrhizae, such as ectomycorrhizae (EC), form structures called Harting nets [3], very similar to what was observed with *M. guizhouense* strain HA11-2 (Figure 1D).

In other species of the genus *Metarhizium*, colonization and the promoting effect on plants such as beans, switchgrass and others have been reported [12,27,34], but this is the first report of this beneficial effect on *M. guizhouense*. Although the relationship between *Metarhizium* and plants has been studied, little is known about the chemical communication between them. Plants release secondary metabolites of high and low molecular mass to the rhizosphere, modifying the microbiota around the roots [2,36]. Some of these molecules released by plants are phytohormones, of which some phytohormones are released to the rhizosphere to establish the mycorrhizal association [1,10,11,14,15,17].

In this study, we evaluated how the fungus is affected by the different phytohormones produced by plants, evaluating germination rate and hyphal length, since these characteristics are important for rapid colonization and establishment of the plant-fungus association [3].

One of the most important groups of phytohormones in plants are GAs, among which gibberellic acid (GA_3_) stands out [37,38]. This family of phytohormones is not only important in plants but also influences fungi. GA_3_ has been found to increase hyphal branching and root colonization in arbuscular mycorrhizal (AM) fungi [39,40]. Here, in contrast to the previously mentioned work, conidial germination and hyphal length were evaluated. Interestingly, a positive change was observed, both in conidial germination rate and hyphal length, up to 30% higher and 2 µm longer than those of the control, respectively, (Figure 2A and Figure 3A).

The phytohormone IAA was the first phytohormone discovered in plants and plays an important role in various processes of plant growth and development [41]. Several microorganisms, both pathogenic and beneficial to plants, including *Metarhizium*, produce different types of auxins. In the case of *Metharizium*, indole 3-acetic acid is produced in medium supplemented with tryptophan [12,42]. Furthermore, a study of AM colonization in tomato plant mutants, with one mutant deficient in auxin signaling and the other with hyperactive polar auxin transport, demonstrating the importance of auxins in symbiosis and the initiation of fungal colonization, showing a strong reduction in colonization rate [43].

Unlike GA_3_, IAA led to an increase in germination rate that was observed only at 6 h (Figure 2B and Supplementary Figure S2), where an increase of 26.81% and an increase in hyphal length of 3.18 µm was observed at 10 h (Figure 3B and Supplementary Figure S3); the average hyphal length was larger in this treatment than in all other treatments. In comparison with the work of Nakamura and Tomita [44,45], where germination and hyphal length of *Neurospora crassa* were evaluated, and compared to that of the control, a positive change of up to 15% was observed for the germination rate with GA_3_, which was 10% higher with IAA [45] and an increase of up to 20 µm for both phytohormones. Interestingly, in this work, the mixture of the two phytohormones enhanced the positive effect on hyphal growth [44].

Strigolactones, which have been studied as a key factor in mycorrhizal association with AM and increased branching of hyphae in EC fungi, are released into the rhizosphere mainly when there is a nutrient deficit and stimulate hyphal growth and branching, increasing physical contact with the root. Thus, AM and EC compensate for the deficiency of these nutrients in the plant [13,21,36,46,47,48]. Synthetic strigolactones with biological activity such as rac-GR24 and other strigolactones, as described in sorghum, have been used; sorgolactone, both with biological activity involved in spore germination, increased the growth and branching of hyphae in AM [21,36,47,49]. As in the aforementioned studies, strigolactones (GR24 and SorL) not only has an effect on AM fungi, but also, as observed here, has a positive effect on *M. guizhouense*. In terms of conidial germination, both strigolactones had a positive effect on germination, GR24 at an early 6-h time and sorgolactone at 8 h (Figure 2C,D), which led to an increase compared to that of the control by up to 24.34% for GR24 and 19.22% for sorgolactone (Supplementary Figure S2). In hyphal growth, the increase was mainly observed at 10 h for both phytohormones (Figure 3C,D), and the trend was clear. For GR24, growth of 3.23 µm more than the control was observed, and for SorL, growth of 1.62 µm more than the control was observed (Supplementary Figure S3). Both phytohormones have been reported to be involved in the colonization process by AM and EC fungi; they also influence the branching of the hyphae of the phytopathogenic fungi *F. oxysporum* and *Botrytis cinerea* [50], and as observed in this study, they also influence the fungus *M. guizhouense* HA11-2. Although the mechanisms by which these phytohormones are detected are not known, it is possible that *Metarhizium* uses mechanisms similar to those of the phytopathogen *Fusarium* because *Metarhizium* is phylogenetically closer to *Fusarium* than to ectomycorrhizal fungi [51].

In the results observed both in the induction of germination and in the promotion of hyphal growth, a greater effect is observed toward the lower concentrations of phytohormones, mainly at concentrations of 1 × 10${}^{-5}$ and 1 × 10${}^{-6}$ M; however, for SorL, the greatest effect is seen for the highest concentration used of 1 × 10${}^{-3}$ at 10 h in both experiments (Figure 2 and Figure 3). This effect is interesting and rules out the possibility of a nutritional effect, since if this were the case, the effect of inducing germination and promoting hyphal growth would be at higher concentrations. In the studies with *Neurospora crassa*, the concentrations of the phytohormones IAA and GA_3_ ranged from 1 × 10${}^{-9}$ to 1 × 10${}^{-4}$ M, where the optimum concentration for the induction of germination was 1 × 10${}^{-4}$ M, both for IAA and GA_3_, while for the induction of hyphal growth was higher using 1 × 10${}^{-6}$ M IAA and 1 × 10^−4^ M GA_3_ [44,45]. For the strigolactones, only the branching of hyphae of the AM fungus *Gigaspora rosea* has been reported, where both GR24 and SorL were used, and the greatest effect was seen at concentrations of 1 × 10${}^{-7}$ and 1 × 10${}^{-5}$, respectively [15]; in addition, the effect was observed with SorL at concentrations of 1 × 10${}^{-15}$ M. The effect of phytohormones at low concentrations reported in fungi and the observed in this work with *M. guizhouense* could be due to the low concentration produced in the plant [52,53,54].

The role of different phytohormones on rhizosphere fungi in modifying them in favor of the plant and establishing the beneficial association for both has been previously determined [36]. This association has been widely studied in AM and EC fungi and other endophytic soil fungi, but scarcely studied in the case of *Metarhizium*, which is also an inhabitant and component in the rhizosphere. Therefore, the elucidation of how phytohormones act on *Metarhizium* is an important part of understanding its relationship with plants.

Knowledge of the communication between plants and *Metarhizium* still has gaps. Recently, how the fungus establishes an association with the plant has begun to be evaluated [24,25,26,27,34]. Here, we try to understand how phytohormones induce growth and germination of the fungus to clarify how the process of its colonization of the plant begins. For example, Awad and collaborators in 2006 and Arens and collaborators in 2013 [18,47] identified that sorghum plants release strigolactones to the soil in concentrations ranging from 10 to 100 pmol per gram of soil, depending on the type of sorghum used, to establish mycorrhizal association with fungi. Other plants, such as corn (*Zea mays*) [18], flaxseed (*Linum usitatissimum* L.) [46,55], cucumber (*Cucumis sativus* L.) [55], lettuce (*Lactuca sativa*) [56], alfalfa (*Medicago sativa*) [57], tobacco (*Nicotiana tabacum*) [58], beans (*Phaseolus vulgaris*), japonica rice (*Oryza japonica*) and tomato (*Solanum lycopersicum*) [36], among other plants of agricultural interest, also produce different types of strigolactones to establish mycorrhizal associations.

In this case, as the first instance for the establishment of root colonization, plants, through the release of phytohormones such as strigolactones, could initiate chemical communication. *Metarhizium* could detect the released strigolactones and germinate faster, as well as induce the growth of mycelium, which can be directed toward the root to establish the association. Although *M. guizhouense* strain HA11-2 is not reported to be endophytic, it is not surprising that it could have this ability since other species, as reported by Sasan and Bidochka in 2012 and Behie and collaborators in 2015 [32,59]. In this way, being between the spaces of the corticoid cells of the plant, the hyphae are in contact with GA_3_ and IAA, where the effect of increased growth of hyphae and rapid colonization was observed. However, looking at it from a biotechnological point of view, when using *Metarhizium* in the field, GA_3_ and IAA could be added externally, helping to establish the beneficial association faster and even increasing the virulence toward the insect, as reported by Liao and collaborators [12].

Given the importance of rapid colonization, here, it was determined that *M. guizohuense* detects the four phytohormones individually, increasing the germination rate and hyphal length. The fact that conidia germinated faster and *Metarhizium* hyphae grew longer, extrapolating from our results, may favor the rapid colonization of plant roots and quickly establish the beneficial association. Given the entomopathogenic capacity of *Metarhizium* [22,35], it can provide extra protection to plant roots against insects and plant pathogenic fungi [60], conferring multiple benefits to the plant, which can be an important factor in its use as a bio tool in agriculture.

## Data Availability

Not applicable.

## Supplementary Materials

The following supporting information can be downloaded at: https://www.mdpi.com/article/10.3390/jof9090945/s1, Figure S1: Brightfield microscopy image of germinated conidia. (A,B) Image of the conidia from the control treatment at 6 h of growth and the treatment with the phytohormone gibberellic acid GA_3_ at a concentration of 1 × 10^−6^ M at 6 h of growth, respectively. The black arrows indicate the germinated conidia. (C,D) Image of the conidia of the control treatment at 10 h of growth and the treatment with the phytohormone strigolactone synthase rac-GR24 (GR24) at a concentration of 1 × 10^−5^ M at 10 h of growth. Red indicates the lengths of the hyphae obtained with the Zeiss Blue edition program. (E,F) Image of the conidia of the control treatment at 10 h of growth and the treatment with the phytohormone gibberellic acid (GA_3_) at a concentration of 1 × 10^−5^ M at 10 h of growth, Figure S2: Heatmap of average germination rates. Phytohormone: GA_3_: gibberellic acid, IAA; 3-indole acetic acid, GR24; synthetic strigolactone rac-GR24, SorL; strigolactone (+−)-sorgolactone. The number indicates the average percentage of germination of each treatment, Figure S3: Heatmap of average hyphal length. Phytohormone: GA_3_: Gibberellic acid, IAA; 3-indole acetic acid, GR24; Strigolactone synthase rac-GR24, SorL; Strigolactone (+−)-sorgolactone. The number indicates the average hyphal length of each treatment, Table S1: Mean germination rate and standard deviation, Table S2: Mean hyphal length and standard deviation.
