# Supplementary material for: Hyphal Growth and Conidia Germination Are Induced by Phytohormones in the Root Colonizing and Plant Growth Promoting Fungus Metarhizium guizhouense
Source: J Fungi (Basel). 2023 Sep 19;9(9):945. doi: 10.3390/jof9090945 (PMC10532501; doi:10.3390/jof9090945)
Supplement: Supplementary file 1 [file jof-09-00945-s001.zip › jof-2577641-supplementary.pdf]

Supplementary material

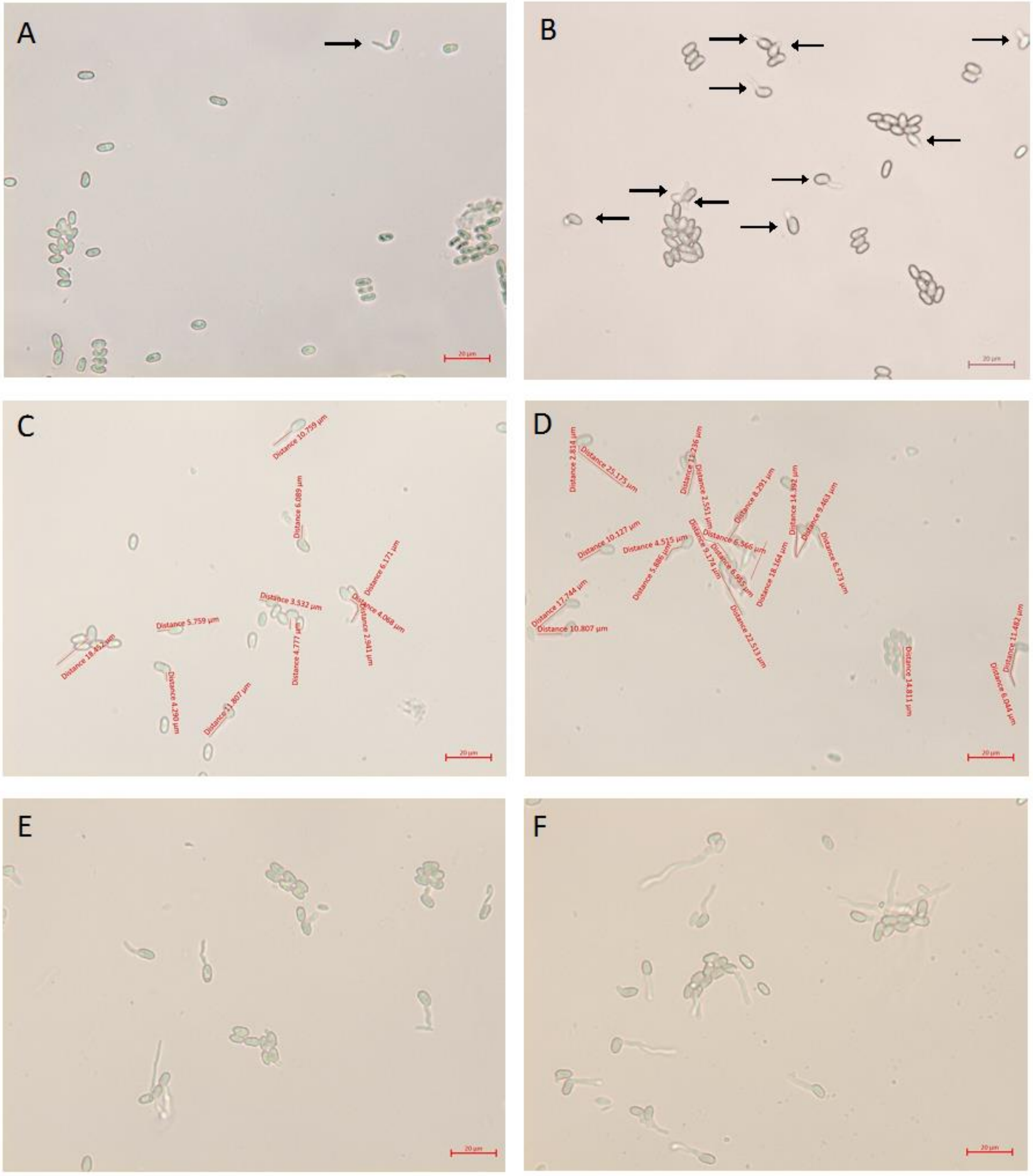

Figure S1. Brightfield microscopy image of germinated conidia. **A and B**:) Image of the conidia from the control treatment at 6 h of growth and the treatment with the phytohormone gibberellic acid GA_3 at a concentration of $1x10^{-6}$ M at 6 h of growth, respectively. The black arrows indicate the germinated conidia. **C and D:)** Image of the conidia of the control treatment at 10 h of growth and the treatment with the phytohormone strigolactone synthase rac-GR24 (GR24) at a concentration of $1x10^{-5}$ M at 10 h of growth. Red indicates the lengths of the hyphae obtained with the Zeiss Blue edition program. **E and F:)** Image of the conidia of the control treatment at 10 h of growth and the treatment with the phytohormone gibberellic acid (GA_3) at a concentration of $1x10^{-5}$ M at 10 h of growth.

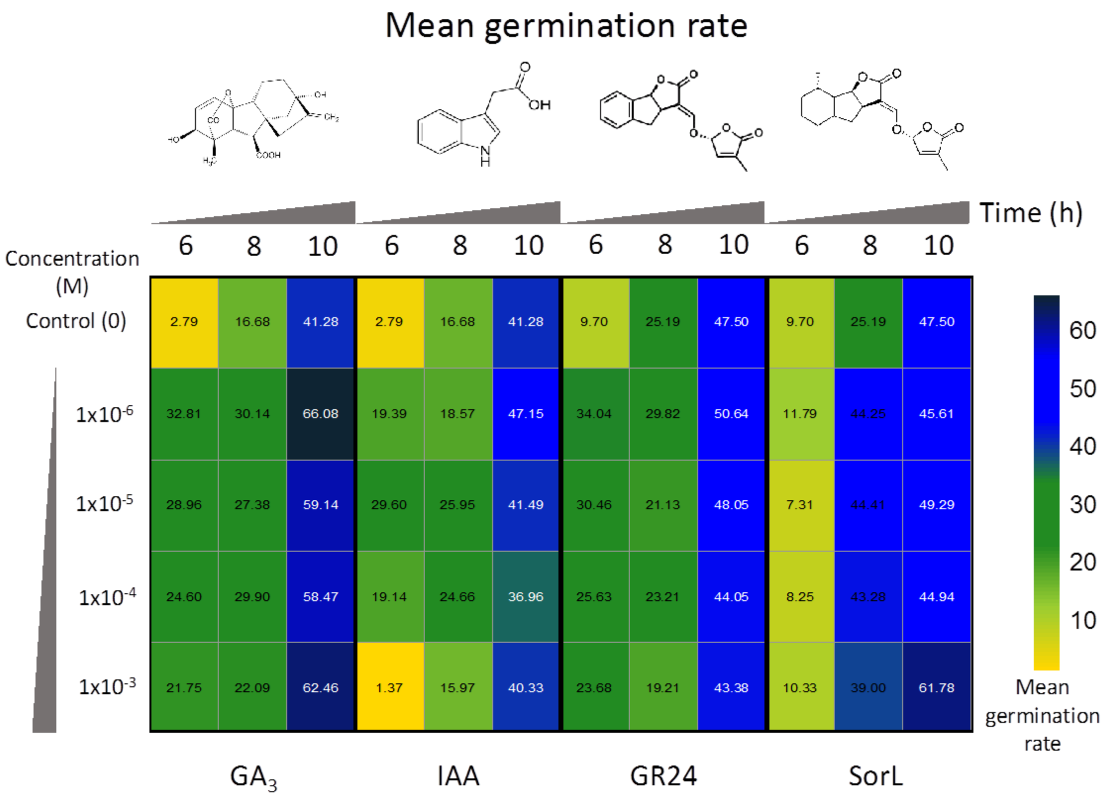

Figure S2. Heatmap of average germination rates. Phytohormone: GA_3: gibberellic acid, IAA; 3-indole acetic acid, GR24; synthetic strigolactone rac-GR24, SorL; strigolactone (+-)-sorgolactone. The number indicates the average percentage of germination of each treatment.

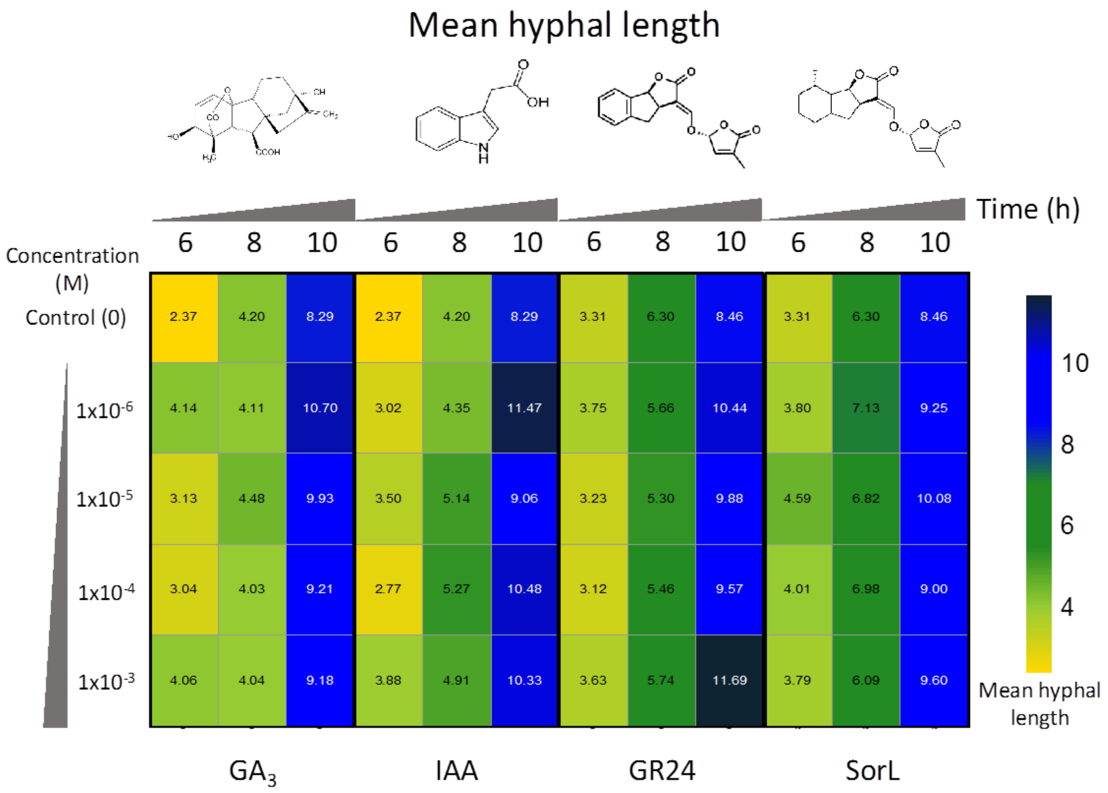

Figure S3. Heatmap of average hyphal length. Phytohormone: GA^3: Gibberellic acid, IAA; 3-indole acetic acid, GR24; Strigolactone synthase rac-GR24, SorL; Strigolactone (+-)-sorgolactone. The number indicates the average hyphal length of each treatment

Table S1. Mean germination rate and standard deviation.

Phytohormone concentration	GA3			IAA			GR24			SorL		
	6h	8h	10h	6h	8h	10h	6h	8h	10h	6h	8h	10h
Control (0 M)	2.793	16.68	41.28	2.793	16.68	41.28	9.695	25.19	47.5	9.695	25.19	47.5
SD Control	3.296	1.732	9.036	3.296	1.732	9.036	6.335	12.95	12.46	6.335	12.95	12.46
$1x10^{-6}$ M	32.81*	30.14*	66.08*	19.39*	18.57	47.15	34.04*	29.82	50.64	11.79	44.25	45.61
SD $1x10^{-6}$ M	11.8	4.344	5.43	6.527	3.044	10.04	9.556	6.907	6.188	2.203	4.554	6.429
$1x10^{-5}$ M	28.96*	27.38*	59.14*	29.6*	25.95*	41.49	30.46*	21.13	48.05	7.312	44.41*	49.29
SD $1x10^{-5}$ M	12.49	5.813	13.03	6.134	4.956	11.21	7.438	12.21	13.9	2.96	10.08	9.894
$1x10^{-4}$ M	24.6*	29.9*	58.47*	19.14*	24.66	36.96	25.63*	23.21	44.05	8.252	43.28*	44.94
SD $1x10^{-4}$ M	12.81	9.721	12.53	14.58	6.665	11.25	7.471	4.898	5.977	4.974	7.335	8.544
$1x10^{-3}$ M	21.75*	22.09	62.46*	1.373	15.97	40.33	23.68*	19.21	43.38	10.33	39*	61.78*
SD $1x10^{-3}$ M	14.06	4.643	8.478	0.3076	7.034	9.822	11.1	7.589	7.526	6.365	5.591	4.555

* Values with significant statistical difference

Table S2. Mean hyphal length and standard deviation

Phytohormone concentration	GA3			IAA			GR24			SorL		
	6h	8h	10h	6h	8h	10h	6h	8h	10h	6h	8h	10h
Control (0 M)	2.367	4.197	8.293	2.367	4.197	8.293	3.311	6.302	8.459	3.311	6.302	8.459
SD Control	1.245	1.934	4.647	1.245	1.934	4.647	1.551	3.081	4.516	1.551	3.081	4.516
$1x10^{-6}$ M	4.143*	4.113	10.7*	3.022	4.348	11.47*	3.746*	5.661	10.44*	3.801	7.131	9.254
SD $1x10^{-6}$ M	1.984	2	4.952	1.334	1.87	6.183	1.746	2.788	6.193	1.544	3.836	5.259
$1x10^{-5}$ M	4.058*	4.04	9.18*	3.883*	4.912	10.33*	3.626	5.744	11.69*	3.792	6.087	9.604*
SD $1x10^{-5}$ M	1.97	1.924	4.488	1.842	2.547	6.04	1.63	2.956	6.604	1.844	3.2	4.89
$1x10^{-4}$ M	3.037*	4.029	9.214*	2.769	5.272*	10.48*	3.123	5.461	9.57	4.011	6.977	9.002
SD $1x10^{-4}$ M	1.367	2.07	4.748	1.152	2.473	5.93	1.242	2.755	5.351	2.153	3.716	4.642
$1x10^{-3}$ M	3.126	4.477	9.928*	3.503*	5.137*	9.063	3.227	5.305	9.88*	4.589*	6.817	10.08*
SD $1x10^{-3}$ M	1.764	2.337	5.268	1.618	2.694	5.397	1.271	2.884	5.439	2.513	3.421	5.117

* Values with significant statistical difference
